# Extensive Atrial Fibrosis and Recalcitrant Atrial Fibrillation: A Case Report and Brief Literature Review

**DOI:** 10.7759/cureus.79169

**Published:** 2025-02-17

**Authors:** Edinen Asuka, Olugbenga Arole, Andrew Ndakotsu

**Affiliations:** 1 Internal Medicine and Preventive Medicine, Griffin Hospital, Derby, USA; 2 Internal Medicine, Griffin Hospital, Derby, USA; 3 Internal Medicine, MedStar Health, Baltimore, USA

**Keywords:** atrial fibrillation, atrial fibrillation ablation, atrial fibrillation recurrence, cardioversion, catheter ablation, electroanatomical mapping, late gadolinium enhancement (lge), maze procedure, recalcitrant atrial fibrillation, speckle tracking echocardiography

## Abstract

Atrial fibrillation is one of the most common supraventricular arrhythmias. It has multiple etiologies, some of which include advanced age, hypertension, valvular heart disease, hyperthyroidism, sleep apnea, ischemic heart disease, cardiomyopathy, and certain medications. Herein, we will discuss a case of extensive atrial fibrosis in a 57-year-old male with recalcitrant atrial fibrillation, the significant role extensive atrial fibrosis plays in the recurrence of atrial fibrillation, a brief pathophysiologic interplay between both pathologies based on current literature and research, and various imaging modalities and treatment options utilized in these cases. Likewise, we will also outline some challenges faced or worth keeping in mind when using the various imaging modalities pertaining to the above subject matter.

## Introduction

Atrial fibrillation is one of the most common cardiac arrhythmias, and it increases the risk of thromboembolic stroke, heart failure, and mortality. Recent studies have explored its association with atrial fibrosis as one of the substrates for its development, persistence, or recurrence. Atrial fibrosis is an intricate and multifactorial pathology that leads to atrial remodeling. Several mechanisms have been linked to this condition, including fibroblast activation, or the transforming growth factor (TGF)-beta pathway, oxidative stress, mechanical stress or stretch-induced fibroblast activation, fibrofatty infiltrations, and inflammatory processes. Recent studies have also mentioned its association with the activation of the coagulation pathway.

Late gadolinium enhancement cardiac magnetic resonance (LGE-CMR) imaging, electroanatomical mapping, and speckle tracking echocardiography (STE) are some of the commonly utilized imaging modalities that are used to evaluate for the presence of significant fibrosis, which can help in guiding treatment plans and possibly prognostication when used effectively and in the right clinical context [1-5]. In addition to the various treatment options available, including pharmacologic therapies and cardioversion, catheter ablation, atrioventricular node ablation with pacemaker placement, or surgical options such as the Maze procedure may be explored, depending on evaluation findings and based on shared decision-making with patients [1-5]. The patient’s clinical context should also be considered when deciding on the treatment plan. 

Herein, this case with a brief literature review underscores certain clinical challenges that may be encountered in patients with recalcitrant atrial fibrillation and underlying extensive atrial fibrosis, diagnostic challenges, and treatment approaches or modalities that can be utilized. 

## Case presentation

**Figure 1:**
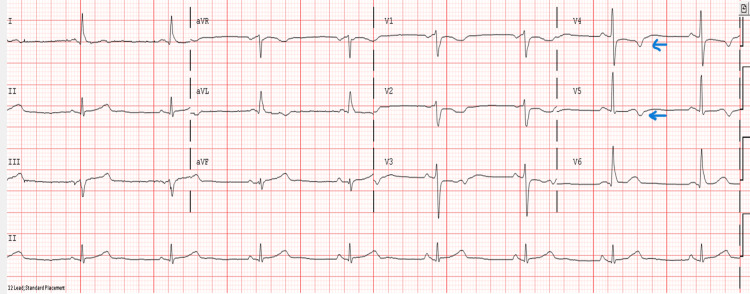
Initial electrocardiogram (ECG) showing sinus rhythm, T wave inversions in lateral precordial leads V4-V5 (with arrows).

A 57-year-old male with a medical history of hypertension, hyperlipidemia, and remote or distant history of gastrointestinal bleeding, said to have occurred once, status post polypectomy and about four years prior, presented with substernal chest discomfort, non-exertional, somewhat tight, no aggravating factors, slightly relieved after receiving medications upon arrival to the emergency department (ED). He endorsed a significant family history of atherosclerotic cardiovascular disease. Significant smoking history, which is about 35 pack years. He was hypertensive with unremarkable physical exam findings at presentation. The initial electrocardiogram (ECG), as depicted in Figure 1, shows a sinus rhythm with new T-wave inversions in lateral precordial leads V4-V5. 

**Figure 2:**
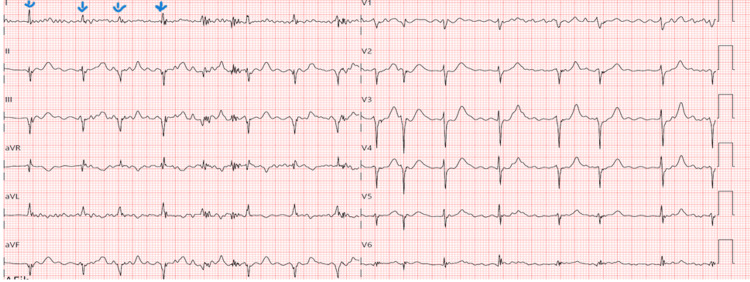
Electrocardiogram (ECG) while on admission showing atrial fibrillation, with irregular R-R intervals (with arrows).

The test results are shown in Table 1: hemoglobin A1c 6.3%, low-density lipoprotein (LDL) cholesterol 87.2 mg/dl, and high-density lipoprotein (HDL) cholesterol 48.8 mg/dl. Initial chest X-ray showed mild diffuse interstitial prominence. He received sublingual (SL) nitroglycerin 0.4 mg x2 at presentation, topical nitroglycerin, intravenous (IV) morphine 4 mg x1, and tab aspirin 162 mg x1. He was subsequently placed on IV heparin per protocol. While on admission, he developed new-onset atrial fibrillation (Figure 2). Thereafter, he remained asymptomatic and clinically stable and underwent an exercise-gated myocardial perfusion scan, which revealed a moderate to large area of predominantly fixed decreased perfusion abnormality along the mid- to basal-lateral and adjacent inferolateral aspects of the left ventricle. He had left cardiac catheterization requiring drug-eluting stent placement in the obtuse marginal branch, about 85% stenosis, with some lesions noted in the diagonal branch of the left anterior descending artery. After shared decision-making with the patient, he was subsequently discharged on apixaban and clopidogrel (initially on triple therapy), atorvastatin, ezetimibe, sacubitril/Valsartan, and carvedilol. After discharge, he underwent cardioversion but later had a recurrence of atrial fibrillation prompting electroanatomical mapping with catheter ablation based on shared decision-making with the patient. Given features indicative of extensive atrial fibrosis during electroanatomical mapping as stated above, this was likely a major contributor to the recurrence of atrial fibrillation in this patient and also a potential risk factor for recurrence despite catheter ablation.

**Table 1:** Test results with laboratory reference range.

Test results	Lab reference range
Hemoglobin A1c: 6.3%	4.2-5.8%
Low-density lipoprotein (LDL) cholesterol: 87.2 mg/dl	65-129 mg/dl
High-density lipoprotein (HDL) cholesterol: 48.8 mg/dl	40-60 mg/dl

## Discussion

Atrial fibrosis is a notable risk factor for severe, recalcitrant atrial fibrillation [1-5]. This patient had recurrence of atrial fibrillation despite cardioversion and was noted to have findings suggestive of extensive atrial fibrosis during electroanatomical mapping prior to his catheter ablation therapy. This appeared to be one of the major factors that contributed to the recalcitrant nature of his atrial fibrillation. In extreme cases, where atrial fibrillation persists or remains recalcitrant despite the above therapies, other treatment strategies such as atrioventricular node ablation and pacemaker placement, rate control strategy, and maze procedure may be explored depending on the clinical context. As earlier stated, atrial fibrosis tends to occur due to the deposition of fibrous tissue within the walls of the atrium, which leads to both structural remodeling and electrophysiological changes that can promulgate the development, persistence, and recurrence of atrial fibrillation [1-5]. 

Based on current literature, atrial fibrosis can occur as a reactive or reparative process. Reactive fibrosis appears to happen in response to inflammatory processes or cardiac pressure overload, which leads to perimysial and endomysial fibrosis; this may occur without the death of cardiomyocytes. Alternatively, reparative fibrosis occurs in response to loss of cardiac myocytes such as in the case of myocardial infarction. Current studies have shown that these processes often entail the release of pro-fibrotic mediators by immune cells, cardiomyocytes, platelets, and activated fibroblasts, which potentiate the activation and proliferation of more fibroblast, as well as differentiation of fibroblasts to secretory myofibroblasts, in addition to other microscopic or cellular changes that tend to occur during these events. These cascades of events lead to collagen deposition, expansion of the extracellular matrix, and eventually atrial fibrosis (atrial remodeling) [1,3,5]. From an electrophysiological standpoint, these foci of fibrosis perpetuate the formation of complex, and disorganized conduction patterns; they also serve as arrhythmogenic substrates for cardiac arrhythmias such as atrial fibrillation. 

It is important to note that while late gadolinium enhancement cardiac magnetic resonance (LGE-CMR) imaging may reasonably reflect areas of atrial scarring or fibrosis, it may miss some areas with endomysial fibrosis [1,3,5]. Also, not all late gadolinium enhancements on cardiac magnetic resonance imaging are indicative of fibrosis. While it may be a strong indicator of scar tissue or fibrosis, especially within an appropriate clinical context, it may also occur secondary to inflammation, amyloid deposition, and tissue edema. Likewise, not all areas with low voltage on electroanatomical mapping are due to atrial fibrosis, factors like enlarged left atrium, other infiltrative heart diseases including amyloid, epicardial fat, and technical issues such as poor catheter contact, or electrode placement and system malfunction may also cause low voltage readings during this procedure. In certain instances, speckle tracking echocardiography is used as well, but inter-observer variability, image quality dependence, software variability, potential artifacts, and lack of direct (fibrous) tissue detection are some of its limitations presently. These are crucial points to keep in mind when using these modalities [1-5].

## Conclusions

This case highlights the need to consider further evaluation of patients with recalcitrant atrial fibrillation for extensive atrial fibrosis, especially in patients with poorly controlled atrial fibrillation, in which case may require prompt initiation of more intensive measures (with or without pharmacologic intervention) like catheter ablation therapy, atrioventricular node ablation with pacemaker placement, and Maze procedure based on shared-decision with the patient while also putting into consideration the patient’s clinical context. Currently, this is often done through the utilization of imaging techniques such as LGE-CMR imaging and electroanatomical mapping. Some studies or literature have also mentioned the use of STE as well, although has its limitations as earlier discussed. While LGE-CMR and electroanatomical mapping may be helpful, it is vital to know that late gadolinium enhancement on LGE-CMR or low-voltage areas on electroanatomical mapping do not always reflect atrial fibrosis. Therefore, consideration of clinical context and apt evaluation while using these modalities is highly recommended. We encourage more robust research on this subject matter, with regard to the enhancement of the precision of these diagnostic and therapeutic modalities, or the development of modalities with higher diagnostic or therapeutic precision. In addition, more research on pharmacological interventions that could target the different pathways and substrates that have been shown to contribute to or lead to atrial remodeling and fibrosis should be explored further. 
